# Self‐Healable and 4D Printable Hydrogel for Stretchable Electronics

**DOI:** 10.1002/advs.202305702

**Published:** 2024-01-23

**Authors:** Huijun Li, Chin Boon Chng, Han Zheng, Mao See Wu, Paulo Jorge Da Silva Bartolo, H. Jerry Qi, Yu Jun Tan, Kun Zhou

**Affiliations:** ^1^ Singapore Centre for 3D Printing, School of Mechanical and Aerospace Engineering Nanyang Technological University 50 Nanyang Avenue Singapore 639798 Singapore; ^2^ Department of Mechanical Engineering, College of Design and Engineering National University of Singapore 9 Engineering Drive Singapore 117575 Singapore; ^3^ School of Mechanical Engineering Georgia Institute of Technology Atlanta GA 30332 USA; ^4^ Centre for Additive Manufacturing National University of Singapore Singapore 117602 Singapore

**Keywords:** 4D printing, electronic, hydrogel, self‐heal, stretchable

## Abstract

Materials with high stretchability and conductivity are used to fabricate stretchable electronics. Self‐healing capability and four‐dimensional (4D) printability are becoming increasingly important for these materials to facilitate their recovery from damage and endow them with stimuli–response properties. However, it remains challenging to design a single material that combines these four strengths. Here, a dually crosslinked hydrogel is developed by combining a covalently crosslinked acrylic acid (AAC) network and Fe^3+^ ions through dynamic and reversible ionically crosslinked coordination. The remarkable electrical sensitivity (a gauge factor of 3.93 under a strain of 1500%), superior stretchability (a fracture strain up to 1700%), self‐healing ability (a healing efficiency of 88% and 97% for the mechanical and electrical properties, respectively), and 4D printability of the hydrogel are demonstrated by constructing a strain sensor, a two‐dimensional touch panel, and shape‐morphing structures with water‐responsive behavior. The hydrogel demonstrates vast potential for applications in stretchable electronics.

## Graphical abstract

A single material is developed for the first time that combines stretchability, conductivity, self‐healing ability, and four‐dimensional (4D) printability. The remarkable stretchability, high electrical sensitivity, self‐healing ability, and 4D printability of the material are demonstrated by constructing a strain sensor, a touch sensor, and shape‐morphing structures with water‐responsive behavior. The developed material demonstrates vast potential for applications in stretchable electronics.

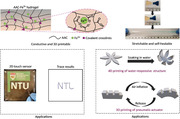

## 1 Introduction

Nowadays, interest in stretchable electronics has grown significantly, driving a need for stretchable materials that can sustain high strain while still fulfilling the requirements of applications such as electronic skin,^[^ ^1^, ^2^ ^]^ soft robotics,^[^ ^3^, ^4^ ^]^ and wearable devices.^[^ ^5^, ^6^ ^]^ Stretchable electronics need to combine high stretchability and conductivity. The self‐healing property of stretchable electronics is also desirable as most of these reported electronics have difficulty in maintaining their performance during long‐term operation due to damage.^[^ ^7^ ^]^ Benefiting from the advantages of three‐dimensional (3D) printing, electronics with complicated shapes can be designed and fabricated. Structures obtained by four‐dimensional (4D) printing can further change their shapes and functions over time, thus showing great potential as stretchable electronics. However, previously reported 4D printable structures were not demonstrated to be conductive and self‐healable. Thus, fabrication of self‐healable and 4D printable electronic devices are of significance to meet the growing demands of escalating multi‐functionality of modern electronics.

Stretchable electronics require two key properties: stretchability and conductivity. However, representative soft electronics fabricated using conductive polymer,^[^ ^8^, ^9^ ^]^ carbon nanotubes,^[^ ^10^, ^11^ ^]^ metal/semiconductor,^[^ ^12^, ^13^ ^]^ and graphene^[^ ^14^ ^]^ demonstrated poor stretchability as they could only sustain a strain of <200%. On the other hand, inspired by the remarkable self‐healing ability of natural organisms that have the capability of autonomous self‐healing after injury, smart self‐healing materials that can self‐repair from physical damage have attracted much attention as they can increase service life.^[^ ^15^, ^16^ ^]^ Much progress has been made on fabrication of self‐healable electronics using self‐healing materials.^[^ ^16^, ^17^, ^18^, ^19^, ^20^ ^]^ Some researchers have 3D printed self‐healable hydrogels with functionality.^[^ ^21^, ^22^ ^]^ Poly (acrylic acid) (AAC) has been widely used for preparing hydrogels. However, the previously reported works focus on investigating the effects of contents of chemicals (i.e., AAC, crosslinker, and metal ions) on the mechanical properties of hydrogels,^[^ ^23^, ^24^ ^]^ modifying the mechanical and conductive properties of AAC‐based hydrogels with additional polymers (i.e., cysteine,^[^ ^25^ ^]^ graphene oxide,^[^ ^26^ ^]^ polyacrylamide^[^ ^27^ ^]^), investigating the self‐healing properties of AAC‐based hydrogel reinforced by additives,^[^ ^28^, ^29^ ^]^ and preparing 3D printable AAC‐based hydrogel with carbon nanotube fillers^[^ ^30^ ^]^ and poly(ethylene glycol).^[31^ ^]^ A single material that combines stretchability, conductivity, self‐healing ability and 4D printability has not been reported as it is an ongoing challenge of designing such multifunctional materials.

For 4D printing of hydrogel structures, two methods, namely extrusion‐based printing^[^ ^32^, ^33^ ^]^ and digital light processing (DLP) printing,^[21^ ^]^ are usually used. Shape‐morphing structures through extrusion‐based printing was developed,^[^ ^32^ ^]^ but the used materials were not demonstrated to be electrically or ionically conductive, and neither they were self‐healing which might limit their functionality and potential applications.

Here, a dually crosslinked AAC–Fe^3+^ hydrogel was synthesized by combining a covalently crosslinked AAC network and Fe^3+^ ions through the dynamic ionically crosslinked metal coordination between the metal ions and carboxylic acid groups in the AAC network. The developed hydrogel exhibited an ultra‐stretchable property, remarkable conductivity, good self‐healing ability, 4D printability, and shape‐memory properties. Additionally, the 4D printing technology and smart AAC–Fe^3+^ hydrogel were integrated to develop soft shape‐morphing structures and soft robotics. This work opens a new avenue for developing hydrogel materials with high stretchability, conductivity, self‐healing ability, and 4D printability.

## 2 Results and Discussion

### 2.1 Concept of AAC–Fe^3+^ Hydrogels

**Figure 1:**
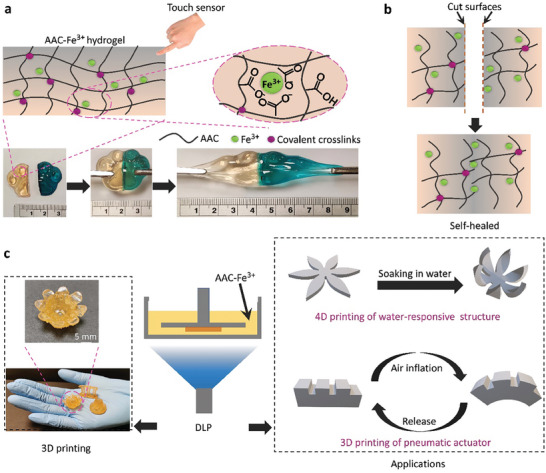
Concept of AAC–Fe^3+^ hydrogels: a) schematic illustration of self‐healing hydrogels which could work as a touch sensor; b) animated self‐healing process between two freshly cut hydrogel pieces at room temperature, and c) images of the 3D printed hydrogel structures and schematic illustration of potential applications of the 3D and 4D printable hydrogel structures using AAC–Fe^3+^ hydrogels.

The dually crosslinked AAC–Fe^3+^ hydrogel was synthesized by combining a covalently crosslinked AAC network and Fe^3+^ ions (**Figure** **1**a). The Fe^3+^ ions served as physical crosslinkers to form ionic coordination interactions with the abundant carboxyl groups of the AAC chains. The obtained hydrogel could be bent, knotted, twisted, stretched in multiple directions, and highly stretched by ≈17 times of its original length, demonstrating its ultrahigh stretchability (Figure S1, Supporting Information). The AAC–Fe^3+^ hydrogel further demonstrated self‐healing properties. In Figure 1a, two freshly cut hydrogel pieces were put into contact for self‐healing at room temperature. After 1 h of contact, the re‐connected part could withstand large stretching, indicating a good self‐healing performance of AAC–Fe^3+^. This was due to the formation of coordination interaction between the metal ions Fe^3+^ and carboxyl groups of AAC (Figure 1b). Hydrogels presented the feature of containing plenty of mobile ions.^[^ ^34^, ^35^ ^]^ Our developed AAC–Fe^3+^ hydrogel was also conductive due to these abundant mobile ions Fe^3+^ in the network. The practical potential of this hydrogel was revealed by its application in sensors.

Benefiting from the advantages of the DLP 3D printing technique, hollow hydrogel structures can be directly fabricated without involving complex manufacturing processes. All the printed objects were displayed to illustrate their delicate structures (Figure 1c). Shape‐morphing structures and a pneumatic actuator based on the AAC–Fe^3+^ hydrogel could also be printed. The shape change (transformation) could be achieved through 4D printing of water‐responsive structures.

### 2.2 Tunable Mechanical Properties of AAC–Fe^3+^ Hydrogels

**Figure 2:**
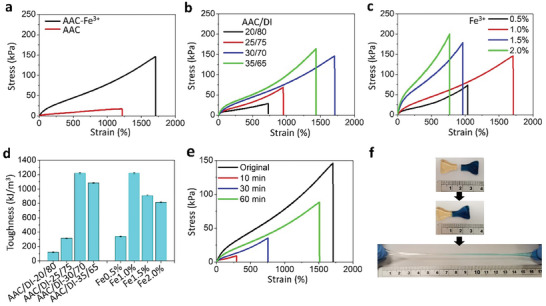
The mechanical properties of AAC–Fe^3+^ hydrogels: a) representative tensile stress–strain curves of the hydrogels; tunable mechanical properties of AAC–Fe^3+^ hydrogels with various weight ratios of AAC to DI water b) and Fe^3+^ ions (c); d) toughness of AAC–Fe^3+^ hydrogels with various weight ratios of AAC to DI and Fe^3+^ ions; e) self‐healing performance of hydrogels under different healing time at room temperature, and f) images of self‐healed hydrogels with ultrahigh stretchability after healing for 60 min.

Chemically crosslinked AAC hydrogels were prepared as the control sample. From the tensile stress–strain curves in **Figure** **2**a, the AAC–Fe^3+^ hydrogel demonstrates superior mechanical properties to the AAC hydrogel by exhibiting a fracture strain ε_b_ of 1708.7% and an ultimate tensile strength (UTS) of 146.6 kPa. Physical bonds (e.g., ionic bonds, hydrogen bonds, and hydrophobic association) are generally weaker than covalent bonds. As a result, in the dually crosslinked AAC–Fe^3+^ hydrogel, the ionic bonds as the sacrificial components were broken first under large deformation, which may establish an efficient energy‐dissipation mechanism to avoid breaking the main framework of the hydrogel. The remarkable stretchability of the dually crosslinked AAC–Fe^3+^ hydrogel was superior to those of the previously reported PAAM‐co‐AAC ionogel,^[^ ^36^ ^]^ agar/poly (N‐hydroxyethyl AAM)‐AAC‐Fe^3+^ hydrogel,^[37^ ^]^ and metal ions‐enhanced AAC hydrogel^[^ ^24^ ^]^ with a breaking strain of 600, 1100, and 1400%, respectively.

To investigate the effect of chemical concentrations on the mechanical properties of the AAC–Fe^3+^ hydrogel, samples were prepared by using different weight ratios of AAC to DI water, Fe^3+^ ions, N, N'‐methylene‐bis‐acrylamide (MBA), and ammonium persulfate (APS). Figure 2b illustrates the effect of the weight ratio of AAC to DI water on the mechanical properties of our developed AAC–Fe^3+^ hydrogel with the concentrations of Fe^3+^ ions, MBA, and APS fixed at 1.0, 0.1, and 1.0 wt.% of that of AAC, respectively. Generally, the mechanical properties of the hydrogel improved as the water content decreased. Both the ε_b_ and UTS increased from 728.9% to 1708.7%, and 27.8 to 146.6 kPa, respectively, with an increasing weight ratio of AAC to DI water from 20/80 to 30/70. A further increase in the weight ratio (e.g., 35/65) resulted in a decrease in ε_b_. Therefore, the weight ratio of AAC to DI water was fixed at 30/70 for subsequent studies.

Shown in Figure 2c, the effect of the content of Fe^3+^ ions on the mechanical properties of our AAC–Fe^3+^ hydrogels were investigated, with the ratio of AAC to DI water fixed at 30/70 and the concentrations of MBA and APS fixed at 0.1 wt% and 1.0 wt% of that of AAC, respectively. Upon increasing the concentrations of Fe^3+^ ions from 0.5 to 1.0 wt.%, ε_b_ sharply increased from 1037.9% to 1708.7% while the UTS increased from 73.7 to 146.6 kPa as the introducing of metal ions could enhance the mechanical property of the hydrogels by forming ionic coordination interactions with carboxyl groups of the AAC chains. However, a further increase in the Fe^3+^ ion content (e.g., 1.5 and 2.0 wt.%) resulted in a hydrogel with weaker mechanical properties, because Fe^3+^ ions had a negative influence on the free radical polymerization, where the massive amount of metal ions might retard the radical polymerization and decrease the molecular weight of poly (AAC).^[^ ^23^, ^38^ ^]^ Thereafter, the Fe^3+^ content was set to be 1.0 wt.%.

The effects of the content of MBA and APS on the mechanical properties of our hydrogel was also investigated (Figure S2, Supporting Information). Although a higher concentration of covalent cross‐linker of MBA represented a higher cross‐linking density of covalently cross‐linked AAC network, Fe^3+^ ions were easier to create a physical network within a sparse AAC network than a dense one.^[^ ^23^ ^]^ Thus, the optimal content of MBA was 0.1 wt.% in this study. The optimal content of APS was 1.0 wt.% as a further increase in the content of APS (e.g., 1.5 wt.%) resulted in a decrease in ε_b_. From Figure 2d, the toughness of AAC–Fe^3+^ hydrogel can be tuned easily by changing the weight ratio of AAC to DI and Fe^3+^ ions content. Additionally, the mechanical performance of the AAC–Fe^3+^ hydrogels under multiple loading‐unloading cycles were measured to investigate their elastic recovery property. As shown in Figure S3 (Supporting Information), the AAC–Fe^3+^ hydrogels were stretched for 50 successive loading‐unloading cycles at a maximum strain of 500%. The results indicated that the AAC–Fe^3+^ hydrogel has a good elastic recovery property, as no obvious plastic strain was observed during the cyclic tensile testing.

The self‐healing properties of our hydrogel under different healing time were investigated at room temperature (Figure 2e). The mechanical performance of the healed sample after 60 min of healing was weaker than that of the original sample according to their stress–strain curves (self‐healing efficiency for the stretchability was ≈88%). Shown in Figure 2f, two freshly cut dog‐bone‐shaped AAC–Fe^3+^ hydrogel pieces were brought into contact. The hydrogel pieces could be lifted immediately after the contact and stretched up to 16 times of its original length after 60 min, indicating the time‐dependent self‐healing behavior of the material. The excellent self‐healing performance of the AAC–Fe^3+^ hydrogel was due to the formation of metal coordination between the Fe^3+^ ions and carboxylic acid groups in the AAC network, and the sticky property of AAC. The healing efficiency and maximum strain of our hydrogel were compared with the other representative self‐healable materials (Table S1, Supporting Information). The results indicated that the proposed AAC–Fe^3+^ hydrogel exhibited a high self‐healing efficiency with an ultra‐stretchability after the self‐healing process.

### 2.3 Electrical Properties of AAC–Fe^3+^ Hydrogels and their Applications

**Figure 3:**
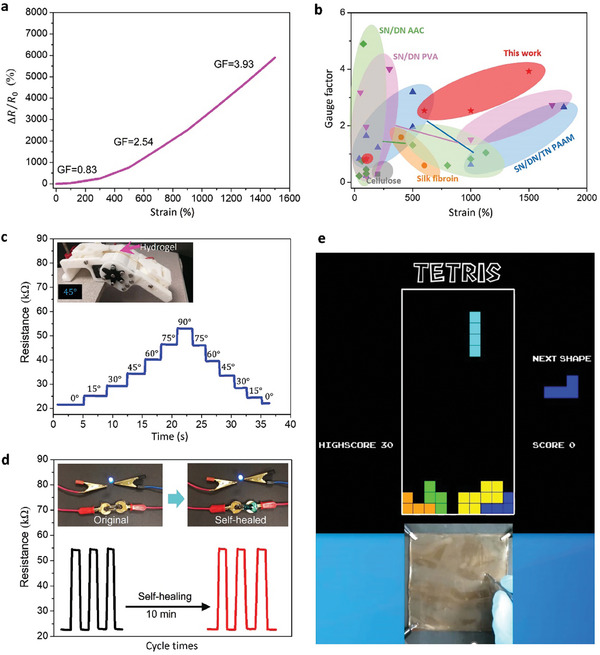
Electrical properties of the hydrogel: a) relative resistance versus tensile strain; b) a comparison between this work and the recently reported hydrogel‐based strain sensors in terms of the GF and strain; c) the real‐time record of the resistance of the hydrogel‐based sensor at different angles; d) the resistance of the original and self‐healed hydrogel‐based sensor at repeated bending angles of 0 and 90 degrees; e) “Tetris” game played by touching different regions of the touch panel to control the directions and movements of the Tetris blocks.

To investigate the electrical sensitivity of the prepared hydrogel, its resistance under different tensile strains (0–1500%) was recorded. The gauge factor (GF), that indicated the electrical sensitivity of a material was calculated by the ratio of the relative resistance change (Δ*R*/*R*_0_ = *R* − *R*_0_/*R*_0_) to the applied strainε, where *R* and *R*_0_ are the resistance with and without the applied strain, respectively. **Figure** **3**a demonstrates the relative resistance change of AAC–Fe^3+^ hydrogel upon stretching. The GF increased slowly and uniformly with the increasing strain. The GF of AAC–Fe^3+^ hydrogel was 0.83 under a strain of 100% and increased to 3.93 as the strain increased to 1500%, indicating a wide strain detection range and a high strain sensitivity. The conductivity of AAC–Fe^3+^ hydrogel was ≈1.22 S m^−1^ (Table S2, Supporting Information), which is superior to that of the reported hydrogels.^[^ ^39^, ^40^, ^41^, ^42^ ^]^

Conventional metal‐based strain sensors have a GF of 2.0, but they show poor stretchability (sustainable strain <5%).^[^ ^43^ ^]^ A stretchable strain sensor had a wide strain detection range of ≈60–200% was reported previously, but it exhibited a small GF of 0.06.^[^ ^11^ ^]^ In order to prepare an ultrasensitive strain sensor with high GF,^[^ ^44^ ^]^ a strain sensor based on ZnSnO_3_ nanowires/microwire was reported, which exhibited an ultra‐high GF of 3740 with a limited strain detection range of 0.35%. The trade‐off between high GF and stretchability of electrical materials caused difficulties in fabricating high‐performance stretchable electronics. Our hydrogel exhibited a high GF of 3.93 and a large strain detection range of up to 1500% compared with the reported representative hydrogel strain sensors (Figure 3b; Table S3, Supporting Information).^[^ ^22^, ^27^, ^45^, ^46^, ^47^, ^48^, ^49^, ^50^, ^51^, ^52^, ^53^, ^54^, ^55^, ^56^, ^57^ ^]^ The performance of our strain sensor was superior to that of the reported hydrogel‐based sensors owing to the ultra‐stretchability and high GF of the AAC‐Fe^3+^ hydrogel.

The performance of our hydrogel as a stretchable electrical material was investigated. The fabricated strain sensor was attached to a robotic bending assembly to detect its bending and stretching motions (Figure S4, Supporting Information). In Figure 3c, the resistance of the strain sensor increased to different levels when the bending angle of the robotic bending assembly increased. The results revealed that the assembled sensor could distinguish the various bending angles of the robotic bending assembly. Moreover, the resistance of this sensor could fall back immediately to its initial level after the robotic bending assembly was relaxed. Our AAC–Fe^3+^ hydrogels are sensitive to bending and stretching motions, and hence could be a promising candidate for fabricating stretchable electronic devices.

To illustrate the self‐healing performance of our AAC–Fe^3+^ hydrogel in terms of its electric properties, the resistance of original and self‐healed samples was investigated. As shown in Figure 3d, the two hydrogels undergoing repeated bending angles of 0 and 90 degrees and their resulting resistance for three cycles were recorded. As expected, the self‐healing efficiency was ≈97%, and the resistance of self‐healed hydrogels were able to almost return to its original value after healing for 10 min at room temperature. A complete circuit comprising a light‐emitting diode (LED) indicator was also used for demonstration the conductivity of the AAC–Fe^3+^ hydrogel. The LED indicator light up when a voltage of 3 V was applied. The hydrogel pieces were healed and worked as a conductor again after the freshly cut surfaces were put back into contact. For practical applications of self‐healable materials, versatile requirements (including short self‐healing time, high self‐healing efficiency, and simple healing procedures) should be satisfied. However, few reported healable materials could meet all the requirements simultaneously.^[^ ^58^ ^]^ Our proposed hydrogels exhibited a superior electrical healing efficiency (≈97%), a short healing time (≈10 min), and a moderate processing condition (without any external stimuli at room temperature), which were superior to those of the poly (N, N'‐dimethylacrylamide)/sillica hydrogels (a healing efficiency of 95% in 15 s),^[59^ ^]^ cellulose nanocomposite hydrogels (a healing efficiency of 88% in 20 min),^[57^ ^]^ composites with carbon nanotubes (a healing efficiency of 95% in 5 min),^[60^ ^]^ nanomaterial/composite polymer (a healing efficiency of ∼100% in 12 h),^[^ ^61^ ^]^ and silver composite polymer (a healing efficiency of 98% in 48 h).^[62^ ^]^ Thus, our proposed hydrogel is a promising candidate as self‐healable sensing material.

AAC–Fe^3+^ hydrogels were used to fabricate a One‐dimensional (1D) touch strip (Figure S5, Supporting Information) and a two‐dimensional (2D) touch panel (Figure S6, Supporting Information) to demonstrate their potential applications. A series of experiments were designed to show the continuous touching performance of the 2D hydrogel touch panel. As shown in Figure S7 (Supporting Information), four electrodes were connected to the four corners of the 2D touch panel and interfaced with a computer through sensing circuits. The signals generated by tracing the “drawing” (input signals) were processed, and another monitor displayed the trace results (output signals). A total of 5 × 5 reference points were used to calibrate the touch point position on the 2D touch panel (Figure S8, Supporting Information). Letters were written on the 2D touch panel (Figure S9, Supporting Information) and the tracing results indicated an excellent match with the pre‐designed pattern and continuous touching performance of the 2D touch panel (Movie S1, Supporting Information). As shown in Figure 3e, a Tetris game was adapted and interfaced with the touch panel to demonstrate the excellent touching performance of the panel. The motion of differently shaped blocks in the Tetris game could be successfully controlled by touching different segments of the panel (Figure S6d and Movie S2, Supporting Information).

### 2.4 4D Printing of AAC–Fe^3+^ Hydrogels and their Applications

**Figure 4:**
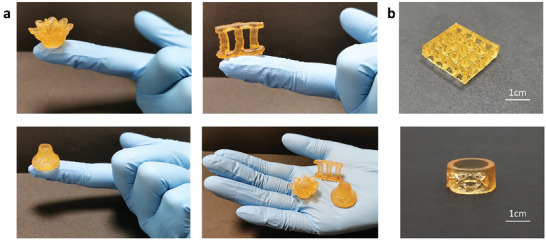
Images of the 3D printed hydrogel structures: a) the printed 3D hydrogel structures (including a complex 3D flower and a hollow vase with a height of 1.5 cm, as well as a model of Marina Bay Sands, a Singapore landmark, with a height of 2.0 cm); b) the other printed hydrogel structures: a 3D grids structure and a ring.

Complex hydrogel structures could be directly fabricated by the DLP 3D printing technique based on our AAC–Fe^3+^ hydrogel. As shown in **Figure** **4**a, a series of hydrogel structures with complex geometries (i.e., a complex 3D flower, a hollow vase and a Singapore landmark) were successfully printed with a high resolution. Each printed 3D structure was put on a finger individually, followed by putting all the printed structures on a hand palm to illustrate their delicate shape and small size. In Figure 4b, the other structures with complex geometries (i.e., the internal porous geometry and hollow geometry) were also successfully printed to demonstrate the 3D printability of the AAC–Fe^3+^ hydrogel. Additionally, the printed 3D structure exhibited a good structural stability as its shape was well maintained for 180 days at room temperature (Figure S10, Supporting Information).

**Figure 5:**
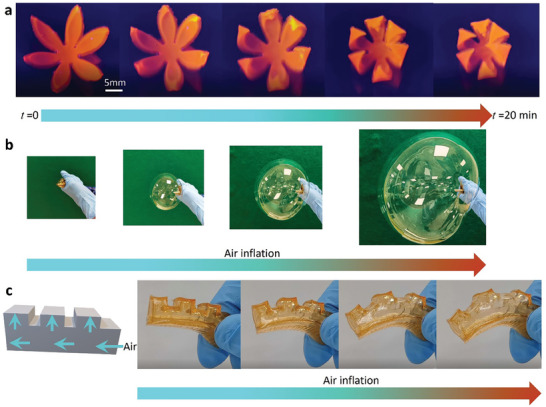
4D printing of AAC–Fe^3+^ hydrogels and their applications: a) photographs of deformation of the water‐responsive flower with gradual closure petals after soaking it in DI water; b) photographs of the air inflation experiment to create a large balloon using a hydrogel sheet; c) schematic of a designed 3D model and its performance of 3D‐printed hydrogels structure as a soft pneumatic actuator.

A series of functional structures were designed to demonstrate the capabilities of 4D printing of AAC–Fe^3+^ hydrogels. To imitate the opening/closing behavior of a flower, petals in a floral form were printed comprised of layers with different crosslinking density, as illustrated in Figure S11 (Supporting Information). After the 3D printed flower was immersed in water, each petal gradually bent and finally generated a closed flower, as shown in **Figure** **5**a (Movie S3, Supporting Information). Harnessing anisotropic swelling induced curvature in bilayer structures.^[^ ^63^ ^]^ The differential swelling between the bottom and top layers could generate curvature as the two layers were forced to remain in contact along the entire midplane. In our printed multi–layered structure, each layer has different swelling as different crosslinking density was applied in each layer. Thus, the resulting water‐responsive structure was due to the break of the top–bottom symmetry, which generated the differential swelling across the thickness similar to the previously reported bilayer structures.^[^ ^64^ ^]^

The shape‐memory behavior of our AAC–Fe^3+^ hydrogel was illustrated by deforming (“programming”) the hydrogel sample at room temperature and soaking it in a Fe^3+^‐ion solution for 30 s, as shown in Figure S12a (Supporting Information). Because of the formation of the metal–ligand coordination interactions between the Fe^3+^ ions and the carboxyl groups from AAC (Figure S12b, Supporting Information), the shape of the hydrogel samples could be temporarily fixed.^[^ ^65^, ^66^ ^]^ The hydrogel sample could recover to its original shape within 210 min through soaking it in a 0.1 wt.% citric acid (CA) solution to remove the Fe^3+^ ions (Figure S13, Supporting Information), which indicated an ion‐triggered shape‐memory behavior. The “programming” and “recovery” steps could be repeated for more than ten times.

The air inflation experiment (Figure 5b) was conducted to demonstrate the stretchability and toughness of the AAC–Fe^3+^ hydrogel. A hydrogel sheet (10 cm × 10 cm × 2 mm) was tightly wrapped around a pipe connected to an air pump. After turning on the air valve, the hydrogel sheet began to expand and form a large balloon, demonstrating its ultrahigh stretchability and remarkable toughness. Inspired by the ultrahigh stretchability of the AAC–Fe^3+^ hydrogel and its capability of forming structures with geometric complexity, a soft actuator with a hollow core was printed (Figure 5c). When the actuator was pressurized, the sawtooth parts expanded and bent toward to the non‐sawtooth side, which caused a driving force to achieve the functionality of a pneumatic actuator.

## 3 Conclusion

A dually crosslinked AAC–Fe^3+^ hydrogel with multiple functions including high stretchability, self‐healing ability, 4D printability, and shape‐memory properties was synthesized. It exhibited several remarkable properties compared with the existing hydrogels, such as a high GF of 3.93 under a strain of 1500% and superior stretchability (a strain of 1700%). Its self‐healing behaviour could be achieved through rebuilding the reversible ionically crosslinked coordination interactions.

The developed hydrogels have potential for versatile applications. Strain and touch sensors were fabricated based on our proposed AAC–Fe^3+^ hydrogel to demonstrate its sensory capability. For example, a soft 2D touch panel with precise touch‐sensing was constructed and its application was successfully demonstrated by writing words and playing the Tetris game. Shape‐morphing structures were designed to demonstrate the capabilities of 4D printing of the AAC–Fe^3+^ hydrogel. This work is the first to illustrate not only ultra‐stretchable and highly conductive properties but also self‐healing ability and 4D printability of a hydrogel for stretchable electronics.

## 4 Experimental Section

### Materials

AAC, MBA, APS, lithium phenyl‐2, 4, 6‐trimethylbenzoylphosphinate (LAP), CA, and iron (III) nitrate nonahydrate (Fe (NO_3_)_3_ • 9H_2_O) were used as received from Sigma–Aldrich, Singapore.

### Hydrogel Preparation

Hydrogels were synthesized by free radical polymerization of solution containing a certain amount of MBA, AAC, APS, and Fe (NO_3_)_3_ • 9H_2_O at room temperature. Briefly, prescribed amounts of AAC and MBA were continuously added in DI water and stirred for 10 min at room temperature. Various mass ratios of AAC to DI water (20/80, 25/75, 30/70, and 35/75) and different concentrations of MBA (0.05, 0.10, 0.15, and 0.20 wt.% with respect to the weight of AAC) were used. After that, Fe (NO_3_)_3_ • 9H_2_O with various concentrations (0.5, 1.0, 1.5, and 2.0 wt.% with respect to the weight of AAC) was gradually added into the mixture under stirring for 10 min followed by gradually adding a certain amount of APS (0.5, 1.0, 1.5, and 2.0 wt.% with respect to the weight of AAC) into the mixture under stirring for 10 min. The obtained mixture was transferred to a petri dish and placed in a UV flood (Shuttered UV system, Epoxy, and equipment technology Pte Ltd) for 3 min to produce the chemical crosslinks at a wavelength of 365 nm.

### Characterization

Tensile tests were performed on the hydrogels by using a tensile tester (Instron 5569; U.K.) with a 100 N load cell at a constant loading rate of 40 mm min^−1^. Samples for tensile tests were prepared with a thickness of 3 mm, a width of 5 mm, and an initial gauge length of 10 mm. Fracture strain ε_b_ and ultimate tensile strength UTS were estimated according to the stress–strain curves obtained from at least six separate tests. Toughness was measured using ImageJ software.

The self‐healing property of the hydrogel was investigated by cutting the samples into two pieces and bringing the freshly cut surfaces in contact with each other without adding any chemicals at room temperature. The mechanical self‐healing efficiency was the proportion of restored mechanical properties. The electrical self‐healing efficiency is the ratio of resistance of the healed to original samples. The average values were calculated from at least six independent samples.

The shape‐memory performance of the AAC–Fe^3+^ hydrogel was investigated by programming the shape of the hydrogel samples under an external force in a solution with 0.1 wt.% of Fe (NO_3_)_3_ • 9H_2_O for 30 s at room temperature to fix their temporary shape. The hydrogel samples were soaked in a solution with 0.1 wt.% of CA for 210 min to recover their original shape.

The hydrogel sample for conductivity testing was cut into circular sheet with a thickness of 3 mm and a diameter of 10 mm. The conductivity was calculated according to equation σ  = *t*/*RA* , where *t*, *R*, and *A* are the thickness, resistance, and cross‐sectional area of a sample, respectively. The average values were calculated from at least six independent samples.

The electrical characteristics of the hydrogel as a strain sensor were analyzed by using a programmable digital multimeter (Keithley, model 2450). Two copper wires connected to the multimeter were attached to both ends of the AAC–Fe^3+^ hydrogel to assemble a strain sensor. The resistance of the strain sensor under different tensile strains was recorded. The strain sensor was attached to a robotic bending assembly to monitor its response (i.e., motion and resistance changes) at different bending angles.

1D hydrogel touch strips (70 mm × 10 mm) were fabricated. Both ends of the hydrogel strip was connected to digital multimeters located at each end of the strip via copper electrodes. A function generator (Model 33210A, Agilent) was connected to both multimeters to create the same phase of alternating current (AC) on either side of the hydrogel strip. The AC voltage of the generator was 1 V with a frequency of 100 Hz. A data acquisition board (NI USB‐9162 with NI 9229 analog input module) and a LabVIEW script were utilized to record and process the signals of the circuit. Each touch position can be determined through comparing the amplitudes of the signal.

A DLP 3D printer (Asiga MAX) with a 385 nm LED light source (18 mW cm^−2^) was used for printing the hydrogel. The ratio of AAC to DI water in the ink for printing was 30/70. The concentrations of MBA, Fe (NO_3_)_3_ • 9H_2_O, and photo‐initiator LAP were 0.1, 1.0, and 0.5 wt.%, respectively, based on the weight of AAC. The time for curing each layer and the layer thickness for printing were set at 10 s and 10 µm, respectively.

## Conflict of Interest

The authors declare no conflict of interest.

## Supporting information

Supporting Information

Supplemental Movie 1

Supplemental Movie 2

Supplemental Movie 3

## Data Availability

The data that support the findings of this study are available from the corresponding author upon reasonable request.
