# Supplementary material for: Self‐Healable and 4D Printable Hydrogel for Stretchable Electronics
Source: Adv Sci (Weinh). 2024 Jan 23;11(13):2305702. doi: 10.1002/advs.202305702 (PMC10987146; doi:10.1002/advs.202305702)
Supplement: Supplementary file 1 — Supporting Information [file ADVS-11-2305702-s002.pdf]

ADVANCED SCIENCE
Open Access

Supporting Information

for *Adv. Sci.*, DOI 10.1002/advs.202305702

Self-Healable and 4D Printable Hydrogel for Stretchable Electronics

Huijun Li, Chin Boon Chng, Han Zheng, Mao See Wu, Paulo Jorge Da Silva Bartolo, H. Jerry Qi, Yu Jun Tan and Kun Zhou**

Supplementary Information

Self-healable and 4D printable hydrogel for stretchable electronics

Huijun Li,[1] Chin Boon Chng,[2] Han Zheng,[1] Mao See Wu,[1] Paulo Jorge Da Silva Bartolo,[1] H. Jerry Qi,[3] Yu Jun Tan,*[2,4] Kun Zhou*[,1]

[1]*Singapore Centre for 3D Printing, School of Mechanical and Aerospace Engineering, Nanyang Technological University, 50 Nanyang Avenue, Singapore 639798, Singapore*

[2]*Department of Mechanical Engineering, College of Design and Engineering, National University of Singapore, 9 Engineering Drive, Singapore 117575, Singapore*

[3]*School of Mechanical Engineering, Georgia Institute of Technology, Atlanta, GA, 30332 USA*

[4]*Centre for Additive Manufacturing, National University of Singapore, Singapore 117602, Singapore*

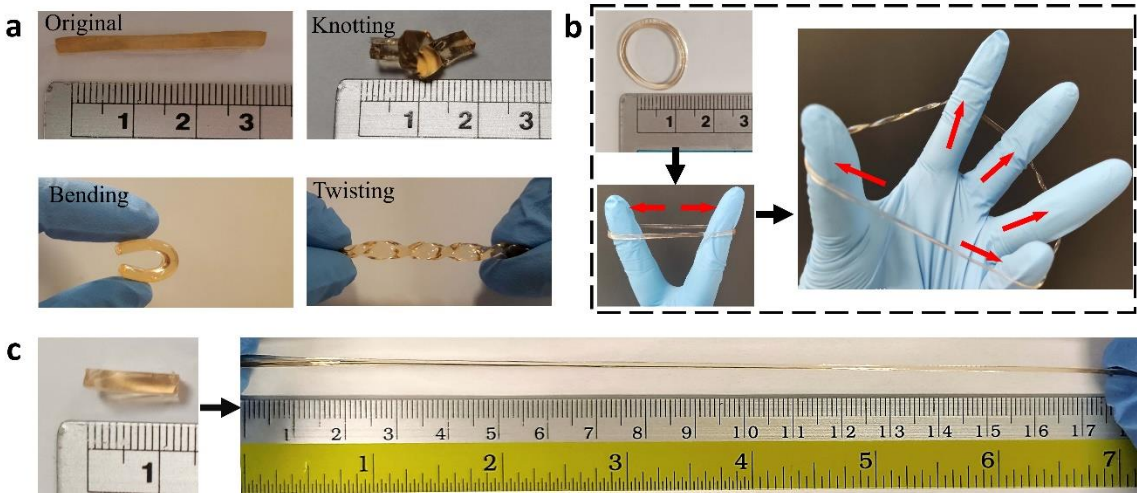

Fig. S1 Photographs of the AAC–Fe^{3+} hydrogel under (a) bending, knotting, twisting, (b) stretching in multiple directions, and (c) stretching under a strain of 1700%.

* Corresponding author. E-mail address: yujun.tan@nus.edu.sg (Y.J. Tan)
* Corresponding author. E-mail address: kzhou@ntu.edu.sg (K. ZHOU)

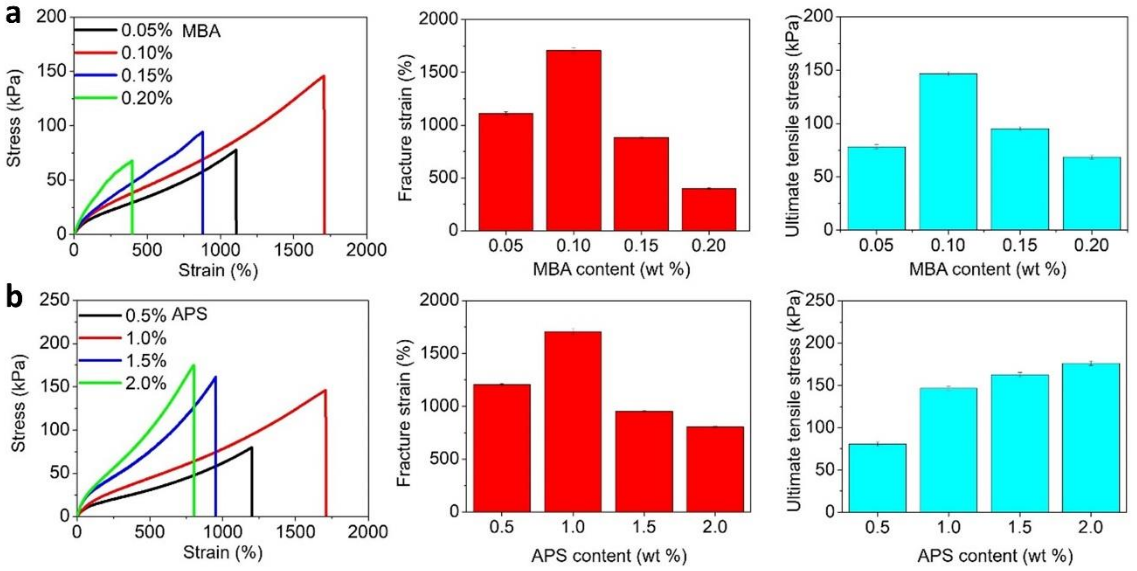

Fig. S2 The tunable mechanical properties of the AAC–Fe^{3+} hydrogel as a function of the concentration of (a) MBA (the ratio of AAC to DI was 30/70 with the concentrations of Fe^{3+} ions and APS fixed at 1.0 and 1.0 wt% of AAC, respectively) and (b) APS (the ratio of AAC to DI was 30/70 with the concentrations of Fe^{3+} ions and MBA fixed at 1.0 and 0.1 wt% of AAC, respectively).

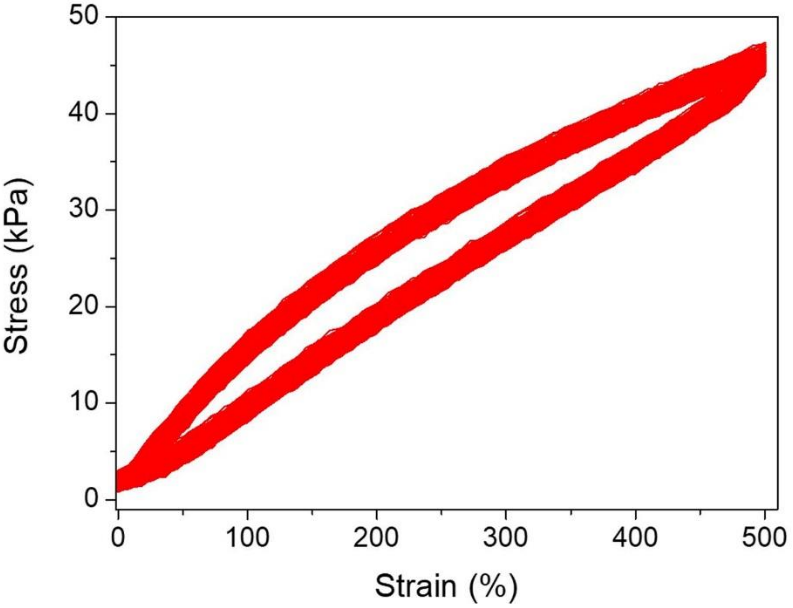

Fig. S3 Mechanical performance of the AAC–Fe^{3+} hydrogel under 50 successive loading-unloading cycles at a maximum strain of 500%.

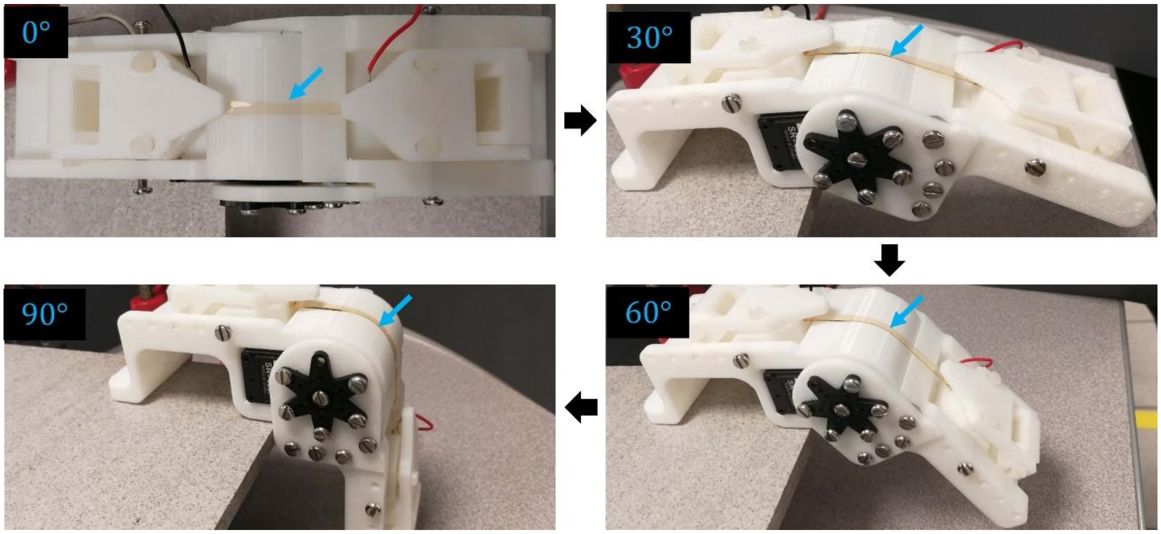

Fig. S4 The hydrogel-based strain sensor was mounted onto a robotic bending assembly to detect its bending motion at representative angles.

Sensing mechanisms of a 1D touch strip

The 1D strip model (**Fig. S5a**) provides a simplified description of a current flowing through the ionic touch sensing system. The current flowed from the electrodes to the grounded human finger through the ionic touch panel when a finger touched the ionic touch strip. The corresponding resistance R_1 and R_2 of the two resistors can be determined by the position of the touch,

$$R_1 = aR \tag{S1}$$

$$R_2 = (1 - a)R \tag{S2}$$

where R is the total resistance of a strip and a is the normalized position. Each resistor was connected to a capacitor of an electrical double layer in series. The impedance Z of two paths can be expressed as

$$Z_1 = R_1 - j\frac{1}{2\pi f C_{\mathrm{EDL}}} \tag{S3}$$

$$Z_2 = R_2 - j\frac{1}{2\pi f C_{\mathrm{EDL}}} \tag{S4}$$

The capacitance per unit area of an electrical double layer C_{EDL} was around $10^{-1} F/m^2$,[1-2] the area of the double layer was around $7 \times 10^{-4}\ m^2$, and the frequency f was 100 Hz. As the resistance of the gel strip was about 20000 Ω, the impedance $Z = 20000\ \Omega - 22.736j \approx 20000.01\angle -0.072$ was approximately equal to the resistance value. Thus, Z_1 and Z_2 were approximately equal to R_1 and R_2, respectively. When a capacitor was added by a finger C_{finger} to the circuit in series, the total current I_{total} flowed through the circuit was

$$I_{total} = \frac{V}{\frac{R_1 R_2}{R_1 + R_2} - j\frac{1}{2\pi f C_{finger}}} \tag{S5}$$

$$I_1 \approx I_{total} \cdot \frac{R_2}{R_1 + R_2} = (1 - a) I_{total} \tag{S6}$$

$$I_2 \approx I_{total} \cdot \frac{R_1}{R_1 + R_2} = a I_{total} \tag{S7}$$

where V is the supply voltage, I_1 and I_2 are the touching currents recorded by the current meter A1 and A2, respectively, I_{total} is the total current where $I_{total} = I_1 + I_2$. Equations (S6) and (S7) could be rearranged into

$$1 - a = \frac{I_1}{I_{total}} \tag{S8}$$

$$a = \frac{I_2}{I_{total}} \tag{S9}$$

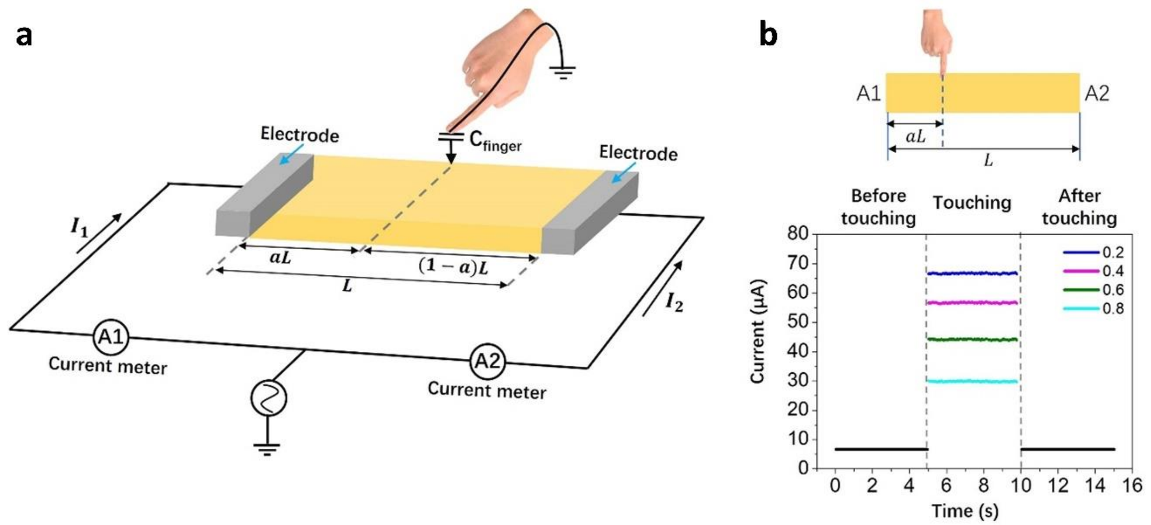

Fig. S5 1D hydrogel touch strip: (a) a schematic showing the working principle of a 1D touch strip during touching. When a finger touches the strip, a closed circuit is formed as the finger is grounded, which allows the current to flow from both sides of the strip to the point of the touch; (b) the current recorded from current meter A1at different touching points (a = 0.2, 0.4, 0.6, or 0.8).

A touching experiment was conducted to find the relationship between the current and touching location. As shown in **Fig. S5b**, the current value recorded from current meter A1changed at different touch points (a = 0.2, 0.4, 0.6, or 0.8). Before each touch, a baseline current in the order of microamperes was detected. The baseline current was a leakage current formed by the parasitic capacitance that generated between the environments and the 1D touch strip.[3] During the touching tests, the current recorded by A1 gradually decreased as the touch position moved to the right side corresponding to an increased value of a. After each touch, the current recorded by A1 returned to the baseline value when the strip was not be touched.

Sensing mechanisms of a 2D touch panel

A 2D hydrogel panel was fabricated to monitor the position of each touch point. Four corners of a rectangular-shaped hydrogel sheet were connected to four current meters via tinned conductive clamps, as shown in **Fig. S6a**. Two normalized distances α and β were used to indicate the position of each touch point on the panel. The bottom left and top right corners of the panel corresponded to $(\alpha, \beta) = (-1, -1)$ and $(\alpha, \beta) = (1, 1)$, respectively. To investigate the sensitivity of the hydrogel to position detection, four points P1, P2, P3, and P4 on the hydrogel were sequentially touched, as shown in **Fig. S6b**. The corresponding current changes recorded from each current meter were displayed against time (**Fig. S6c**). For example, when point P1 was touched, the closest current meter A1 demonstrated the largest current value, while the farthest current meter A3 showed the lowest current value. In the 2D case, the current measured from the four current meters was proportional to the proximity of the touch point to each electrode, which was similar to that in the 1D case. The Tetris game was played to show the touching performance of the 2D touch panel, where the motion of differently shaped blocks could be successfully controlled by touching different segments of the touch panel (**Fig. S6d**).

A sensing system (including a controller board, NI Labview, Python calibration script and Python mapping script) was developed to interface the 2D hydrogel touch panel with a computer screen used to display the output results (**Fig. S7**). The position of a touch point could be estimated according to the following equations [4-5]

$$\alpha \propto \frac{I_2 + I_3}{I_1 + I_2 + I_3 + I_4} \tag{S10}$$

$$\beta \propto \frac{I_1 + I_2}{I_1 + I_2 + I_3 + I_4} \tag{S11}$$

where I_1, I_2, I_3, and I_4 are currents recorded from current meters A1, A2, A3, and A4, respectively. In total 5 × 5 reference points were used to calibrate the touch point position on the 2D touch panel (**Fig. S8**).

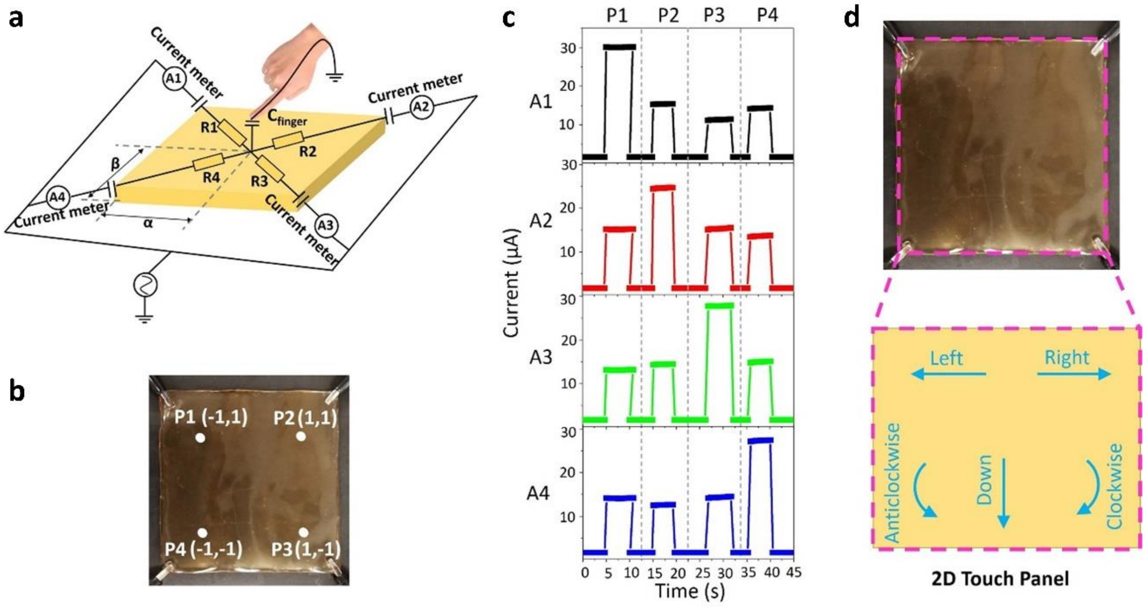

Fig. S6 2D hydrogel touch panel: (a) a schematic showing the working principle of a 2D hydrogel touch panel, which uses two normalized distances (α and β) to indicate the touching position; (b) four points (P1 to P4) on the hydrogel touch panel are touched sequentially and the corresponding readings of the current meters are plotted against time (c) to investigate the sensitivity of the hydrogel to position detection; (d) the surface of the touch panel was divided into five segments to control the block motions (including moving left, right and down, and rotating clockwise and anticlockwise).

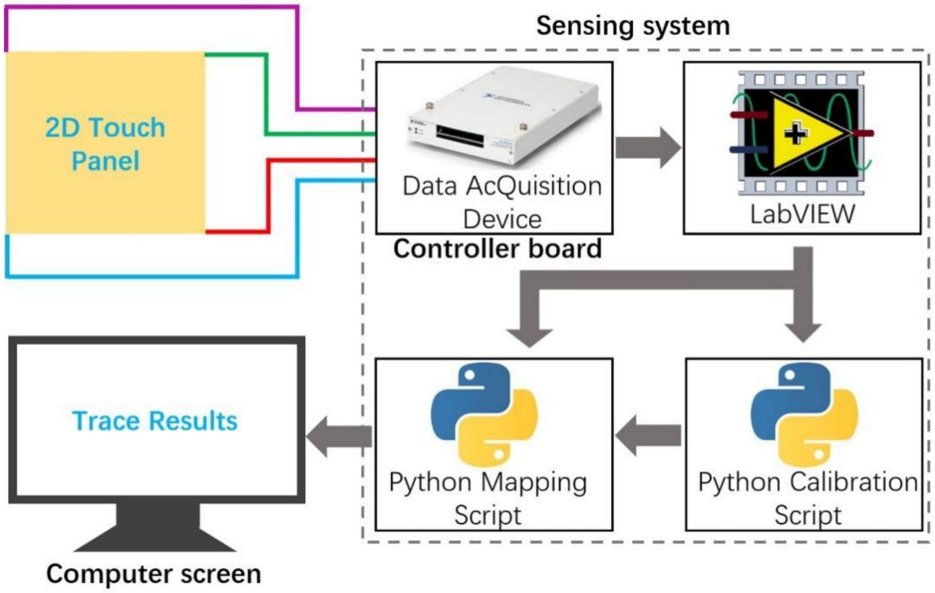

Fig. S7 A schematic showing the communication between a 2D hydrogel touch panel and a computer screen. The 2D hydrogel touch panel was connected to a sensing system that was used to process the acquired signal and the output results were displayed on the computer screen.

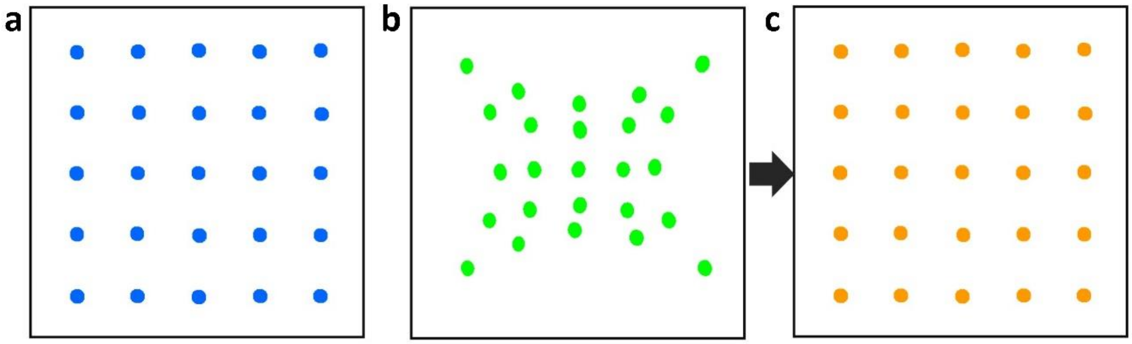

Fig. S8 Calibration process of a 2D hydrogel touch panel. (a) A schematic showing 5 × 5 reference points used to calibrate the position of touch points on the 2D touch panel. Detected results of the references points (b) and calibrated results (c) that showing an acceptable touch signal by using piecewise-affine transformation on the touch panel.

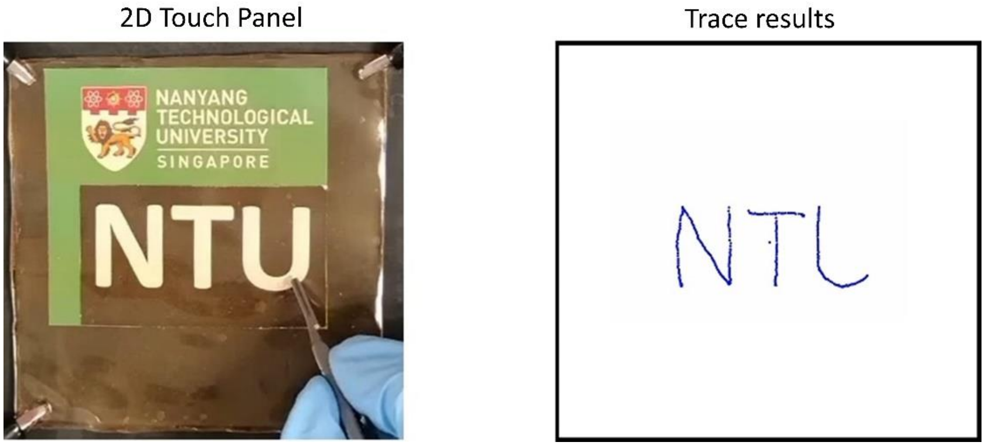

Fig. S9 Demonstration of writing words on a 2D hydrogel touch panel to illustrate its touch-sensing performance.

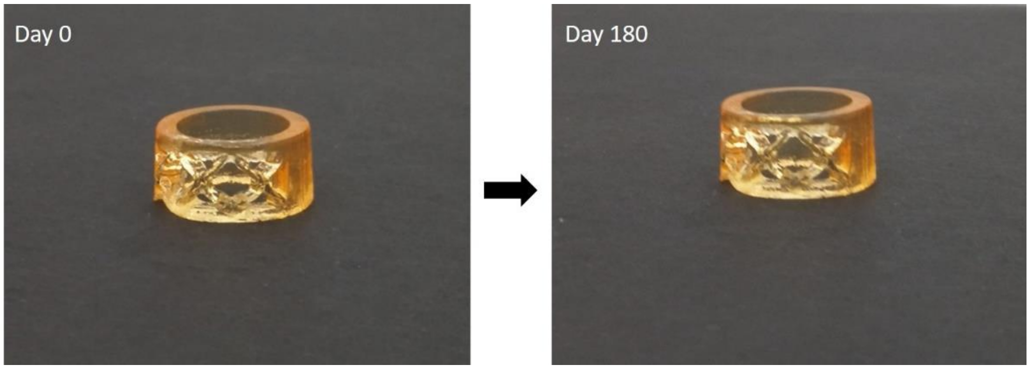

Fig S10. Images demonstrating structural stability of the printed structure over 180 days at room temperature.

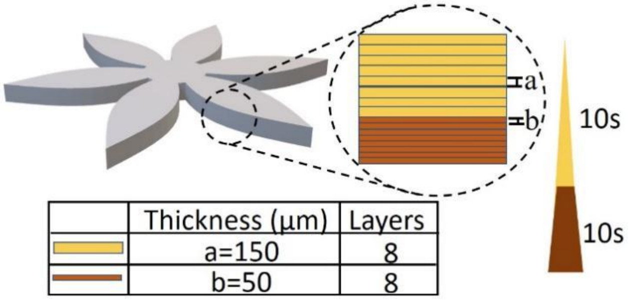

Fig. S11 Schematic of a water-responsive flower with different crosslinking densities across its thickness.

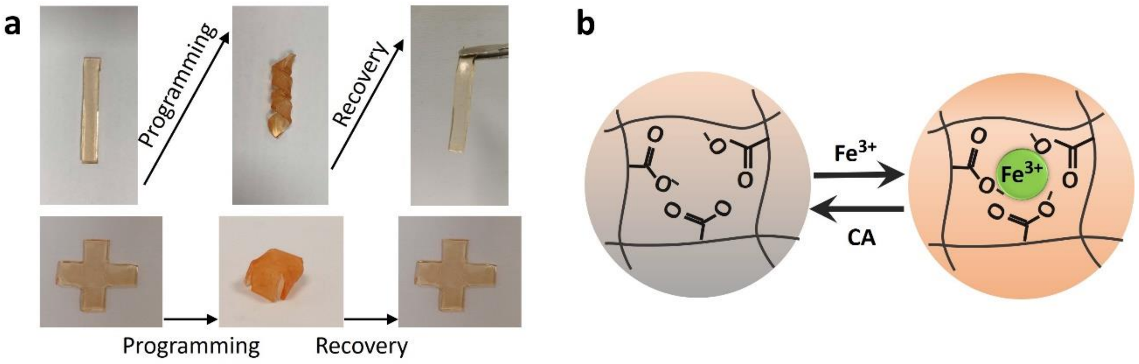

Fig. S12 The shape-memory behavior of AAC–Fe^{3+} hydrogel: (a) images of metal ions-induced shape-memory behaviors of a spiral and cubic structure and (b) the mechanism of their shape-memory behaviors.

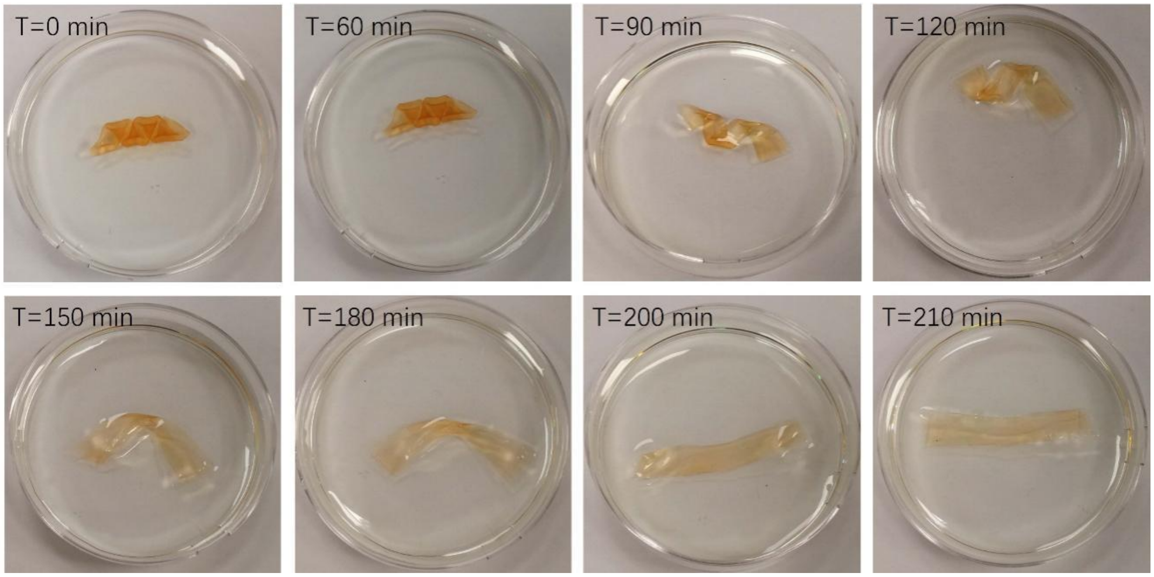

Fig. S13 Photographs demonstrating the shape-recovery process of the AAC–Fe^{3+} hydrogel in a citric acid solution.

Table S1. Performances of recently reported self-healable materials.

Self-healing materials	Maximum strain of healed sample (%)	Healing efficiency (%)	Ref.
Gelatin/PAAM	1170<br>280	29.3 (40h)<br>6.9 (0.5h)	[6]
k-carrageenan/PAAM	100	5.8	[7]
PHEMA-SWCNT	292.5	90	[8]
Silicon/carbon	270	90	[9]
Graphene/silk	115	100	[10]
Dually crosslinked AAC	~1800<br>~1600<br>~1000	~93 (12h)<br>~81 (6h)<br>54 (2h)	[11]
AAC/ Fe^{3+}	286	88	[12]
AAC/silica	1800	78	[13]
AAC/PPy/chitosan/ Fe^{3+}	1500	100	[14]
AAC–Fe^{3+}	1600	88 (1h)	This work

Table S2. Resistance and conductivity of the hydrogel samples.

Sample number	Resistance (Ω)	Conductivity (S m^{-1})
1	7.66	1.24
2	8.03	1.19
3	7.85	1.22
4	7.93	1.20
5	7.69	1.24
6	7.81	1.22

Table S3. Performances of recently reported hydrogel strain sensors.

Strain sensor	Strain (%)	Gauge factor	Ref.
Cellulose ionic hydrogel	220	0.297	15
Silk fibroin-based hydrogel	50	0.8	16
	400	1.6	
	600	0.6	
k-carrageenan/PAAm DN hydrogel	1000	0.63	17
	100	0.23	
PAAm/PAA–Fe^{3+}/NaCl hydrogel	200	1.23	18
	500	1.96	
NaCl/ SA/PAAm hydrogel	1800	2.66	19
HPAAm/CS-MWCNT hybrid hydrogel	100	1.65	20
	500	3.2	
PAAm/LiCl/PDMS hydrogel	40	0.84	21
PVA organo-hydrogel	1700	2.74	22
	100	0.83	

PVA/glycerol/NaCl ionic hydrogel	0~100	1.96	23
	100~300	4.01	
TA–CNT–glycerol–PVA hydrogel	50	3.18	24
SWCNT/PVA hydrogel	100	0.24	25
	1000	1.51	
AAC/rGO/Fe^{3+} hydrogel	100	0.31	26
	500	1.32	
AAC/PANI hydrogel	0-800	0.6	27
	800-1130	1.05	
agar/AAC/Fe^{3+} DN hydrogel	100	0.46	28
	1000	0.83	
AAC/TA@CNC hydrogel	0-40	0.23	29
	40-65	0.76	
	65-75	4.9	
AAC–Fe^{3+} hydrogel	100	0.83	This work
	600	2.54	
	1500	3.93	

References

(1) Keplinger, C.; Sun, J.-Y.; Foo, C. C.; Rothemund, P.; Whitesides, G. M.; Suo, Z. Stretchable, Transparent, Ionic Conductors. *Science* **2013,** *341* (6149), 984-987.

(2) Lee, K. H.; Kang, M. S.; Zhang, S.; Gu, Y.; Lodge, T. P.; Frisbie, C. D. "Cut and Stick" Rubbery Ion Gels as High Capacitance Gate Dielectrics. *Advanced Materials* **2012,** *24* (32), 4457-4462.

(3) Haga, H.; Yanase, J.; Kamon, Y.; Takatori, K.; Asada, H.; Kaneko, S. Touch Panel Embedded IPS-LCD with Parasitic Current Reduction Technique. *SID Symposium Digest of Technical Papers* **2010,** *41* (1), 669-672.

(4) William Pepper, J. U.S. patent 4293734, 1981.

(5) Han, C.; Yang, F.; Guo, X.; Bai, Y.; Liu, G.; Sun, H.; Wang, P.; Liu, W.; Wang, R. Ultra-Stretchable Self-Healing Composite Hydrogels as Touch Panel. *Advanced Materials Interfaces* **2021,** *8* (18), 2100742.

(6) Yan, X.; Chen, Q.; Zhu, L.; Chen, H.; Wei, D.; Chen, F.; Tang, Z.; Yang, J.; Zheng, J. High strength and self-healable gelatin/polyacrylamide double network hydrogels. *Journal of Materials Chemistry B* **2017,** *5* (37), 7683-7691.
(7) Liu, S.; Li, L. Recoverable and Self-Healing Double Network Hydrogel Based on κ-Carrageenan. *ACS Appl. Mater. Interfaces* **2016,** *8* (43), 29749-29758.
(8) Guo, K.; Zhang, D.-L.; Zhang, X.-M.; Zhang, J.; Ding, L.-S.; Li, B.-J.; Zhang, S. Conductive Elastomers with Autonomic Self-Healing Properties. *Angewandte Chemie International Edition* **2015,** *54* (41), 12127-12133.
(9) Wang, C.; Wu, H.; Chen, Z.; McDowell, M. T.; Cui, Y.; Bao, Z. Self-healing chemistry enables the stable operation of silicon microparticle anodes for high-energy lithium-ion batteries. *Nature Chemistry* **2013,** *5* (12), 1042-1048.
(10) Wang, Q.; Ling, S.; Liang, X.; Wang, H.; Lu, H.; Zhang, Y. Self-Healable Multifunctional Electronic Tattoos Based on Silk and Graphene. *Advanced Functional Materials* **2019,** *29* (16), 1808695.
(11) Zhong, M.; Liu, Y.-T.; Liu, X.-Y.; Shi, F.-K.; Zhang, L.-Q.; Zhu, M.-F.; Xie, X.-M. Dually cross-linked single network poly(acrylic acid) hydrogels with superior mechanical properties and water absorbency. *Soft Matter* **2016,** *12* (24), 5420-5428.
(12) Wei, Z.; He, J.; Liang, T.; Oh, H.; Athas, J.; Tong, Z.; Wang, C.; Nie, Z. Autonomous Self-Healing of Poly(acrylic acid) Hydrogels Induced by The Migration of Ferric Ions. *Polym. Chem.* **2013,** *4* (17), 4601-4605.
(13) Zhong, M.; Liu, X.-Y.; Shi, F.-K.; Zhang, L.-Q.; Wang, X.-P.; Cheetham, A. G.; Cui, H.; Xie, X.-M. Self-healable, tough and highly stretchable ionic nanocomposite physical hydrogels. *Soft Matter* **2015,** *11* (21), 4235-4241.
(14) Darabi, M. A.; Khosrozadeh, A.; Mbeleck, R.; Liu, Y.; Chang, Q.; Jiang, J.; Cai, J.; Wang, Q.; Luo, G.; Xing, M. Skin-Inspired Multifunctional Autonomic-Intrinsic Conductive Self-Healing Hydrogels with Pressure Sensitivity, Stretchability, and 3D Printability. *Advanced Materials* **2017,** *29* (31), 1700533.
(15) Tong, R.; Chen, G.; Pan, D.; Tian, J.; Qi, H.; Li, R. a.; Lu, F.; He, M. Ultrastretchable and Antifreezing Double-Cross-Linked Cellulose Ionic Hydrogels with High Strain Sensitivity under a Broad Range of Temperature. *ACS Sustainable Chemistry & Engineering* **2019,** *7* (16), 14256-14265.
(16) He, F.; You, X.; Gong, H.; Yang, Y.; Bai, T.; Wang, W.; Guo, W.; Liu, X.; Ye, M. Stretchable, Biocompatible, and Multifunctional Silk Fibroin-Based Hydrogels toward Wearable Strain/Pressure Sensors and Triboelectric Nanogenerators. *ACS Applied Materials & Interfaces* **2020,** *12* (5), 6442-6450.
(17) Liu, S.; Li, L. Ultrastretchable and Self-Healing Double-Network Hydrogel for 3D Printing and Strain Sensor. *ACS Appl. Mater. Interfaces* **2017,** *9* (31), 26429-26437.
(18) Li, S.; Pan, H.; Wang, Y.; Sun, J. Polyelectrolyte complex-based self-healing, fatigue-resistant and anti-freezing hydrogels as highly sensitive ionic skins. *Journal of Materials Chemistry A* **2020,** *8* (7), 3667-3675.

(19) Zhang, X.; Sheng, N.; Wang, L.; Tan, Y.; Liu, C.; Xia, Y.; Nie, Z.; Sui, K. Supramolecular nanofibrillar hydrogels as highly stretchable, elastic and sensitive ionic sensors. *Materials Horizons* **2019,** *6* (2), 326-333.
(20) Xia, S.; Song, S.; Jia, F.; Gao, G. A flexible, adhesive and self-healable hydrogel-based wearable strain sensor for human motion and physiological signal monitoring. *Journal of Materials Chemistry B* **2019,** *7* (30), 4638-4648.
(21) Tian, K.; Bae, J.; Bakarich, S. E.; Yang, C.; Gately, R. D.; Spinks, G. M.; in het Panhuis, M.; Suo, Z.; Vlassak, J. J. 3D Printing of Transparent and Conductive Heterogeneous Hydrogel–Elastomer Systems. *Advanced Materials* **2017,** *29* (10), 1604827.
(22) Dong, X.; Guo, X.; Liu, Q.; Zhao, Y.; Qi, H.; Zhai, W. Strong and Tough Conductive Organo-Hydrogels via Freeze-Casting Assisted Solution Substitution. *Advanced Functional Materials n/a* (n/a), 2203610.
(23) Pan, S.; Xia, M.; Li, H.; Jiang, X.; He, P.; Sun, Z.; Zhang, Y. Transparent, high-strength, stretchable, sensitive and anti-freezing poly(vinyl alcohol) ionic hydrogel strain sensors for human motion monitoring. *Journal of Materials Chemistry C* **2020,** *8* (8), 2827-2837.
(24) He, P.; Wu, J.; Pan, X.; Chen, L.; Liu, K.; Gao, H.; Wu, H.; Cao, S.; Huang, L.; Ni, Y. Anti-freezing and moisturizing conductive hydrogels for strain sensing and moist-electric generation applications. *Journal of Materials Chemistry A* **2020,** *8* (6), 3109-3118.
(25) Cai, G.; Wang, J.; Qian, K.; Chen, J.; Li, S.; Lee, P. S. Extremely Stretchable Strain Sensors Based on Conductive Self-Healing Dynamic Cross-Links Hydrogels for Human-Motion Detection. *Advanced Science* **2017,** *4* (2), 1600190.
(26) Jing, X.; Mi, H.-Y.; Peng, X.-F.; Turng, L.-S. Biocompatible, self-healing, highly stretchable polyacrylic acid/reduced graphene oxide nanocomposite hydrogel sensors via mussel-inspired chemistry. *Carbon* **2018,** *136*, 63-72.
(27) Wang, Z.; Zhou, H.; Lai, J.; Yan, B.; Liu, H.; Jin, X.; Ma, A.; Zhang, G.; Zhao, W.; Chen, W. Extremely stretchable and electrically conductive hydrogels with dually synergistic networks for wearable strain sensors. *Journal of Materials Chemistry C* **2018,** *6* (34), 9200-9207.
(28) Li, H.; Zheng, H.; Tan, Y. J.; Tor, S. B.; Zhou, K. Development of an Ultrastretchable Double-Network Hydrogel for Flexible Strain Sensors. *ACS Applied Materials & Interfaces* **2021,** *13* (11), 12814-12823.
(29) Shao, C.; Wang, M.; Meng, L.; Chang, H.; Wang, B.; Xu, F.; Yang, J.; Wan, P. Mussel-Inspired Cellulose Nanocomposite Tough Hydrogels with Synergistic Self-Healing, Adhesive, and Strain-Sensitive Properties. *Chemistry of Materials* **2018,** *30* (9), 3110-3121.
